# Effect of phosphorus supply on root traits of two *Brassica oleracea* L. genotypes

**DOI:** 10.1186/s12870-020-02558-2

**Published:** 2020-08-05

**Authors:** Paula Pongrac, Hiram Castillo-Michel, Juan Reyes-Herrera, Robert D. Hancock, Sina Fischer, Mitja Kelemen, Jacqueline A. Thompson, Gladys Wright, Matevž Likar, Martin R. Broadley, Primož Vavpetič, Primož Pelicon, Philip J. White

**Affiliations:** 1 Ecological Science Group, The James Hutton Institute, Invergowrie, Dundee, DD2 5DA UK; 2 Jožef Stefan Institute, Jamova 39, SI-1000 Ljubljana, Slovenia; 3 European Synchrotron Radiation Facility, Grenoble, France; 4 Cell and Molecular Sciences, The James Hutton Institute, Invergowrie, Dundee, DD2 5DA UK; 5 Future Food Beacon of Excellence and the School of Biosciences, University of Nottingham, Nottingham, LE12 5RD UK; 6 Jožef Stefan International Postgraduate School, Jamova 39, SI-1000 Ljubljana, Slovenia; 7 Biotechnical Faculty, University of Ljubljana, Jamnikarjeva 101, SI-1000 Ljubljana, Slovenia; 8 Plant and Crop Sciences Division, University of Nottingham, Loughborough, LE12 5RD UK; 9 Distinguished Scientist Fellowship Program, King Saud University, Riyadh, 11451 Saudi Arabia; 10 College of Resources and Environment, Huazhong Agricultural University, Wuhan, 430070 China

**Keywords:** Phosphorus use efficiency, Phosphorus deficiency, Root exudates, Spatial distribution of phosphorus, Kale, Broccoli, RNAseq, Gene expression

## Abstract

***Background*:**

Phosphorus (P) deficiency limits crop production worldwide. Crops differ in their ability to acquire and utilise the P available. The aim of this study was to determine root traits (root exudates, root system architecture (RSA), tissue-specific allocation of P, and gene expression in roots) that (a) play a role in P-use efficiency and (b) contribute to large shoot zinc (Zn) concentration in *Brassica oleracea*.

***Results*:**

Two *B. oleracea* accessions (var. *sabellica* C6, a kale, and var. *italica* F103, a broccoli) were grown in a hydroponic system or in a high-throughput-root phenotyping (HTRP) system where they received *Low P* (0.025 mM) or *High P* (0.25 mM) supply for 2 weeks. In hydroponics, root and shoot P and Zn concentrations were measured, root exudates were profiled using both Fourier-Transform-Infrared spectroscopy and gas-chromatography-mass spectrometry and previously published RNAseq data from roots was re-examined. In HTRP experiments, RSA (main and lateral root number and lateral root length) was assessed and the tissue-specific distribution of P was determined using micro-particle-induced-X-ray emission. The C6 accession had greater root and shoot biomass than the F103 accession, but the latter had a larger shoot P concentration than the C6 accession, regardless of the P supply in the hydroponic system. The F103 accession had a larger shoot Zn concentration than the C6 accession in the *High P* treatment. Although the F103 accession had a larger number of lateral roots, which were also longer than in the C6 accession, the C6 accession released a larger quantity and number of polar compounds than the F103 accession. A larger number of P-responsive genes were found in the *Low P* treatment in roots of the F103 accession than in roots of the C6 accession. Expression of genes linked with “phosphate starvation” was up-regulated, while those linked with iron homeostasis were down-regulated in the *Low P* treatment.

**Conclusions:**

The results illustrate large within-species variability in root acclimatory responses to P supply in the composition of root exudates, RSA and gene expression, but not in P distribution in root cross sections, enabling P sufficiency in the two *B. oleracea* accessions studied.

## Background

In plants, phosphorus (P) is involved in cellular bioenergetics and metabolic regulation, and is an important structural component of essential biomolecules including DNA, RNA, phospholipids, ATP and sugar-phosphates [1, 2]. When P is in limited supply plants utilise a multifaceted set of morphological, biochemical and molecular strategies to increase the availability of P in the soil, adjust root growth to access P-rich soil patches and / or modify the ways in which P is used internally (improving P-acquisition, P-reallocation and P-remobilisation within the plant) [1, 3–5].

The initial biochemical and morphological adjustments made by plants to increase the acquisition of P occur in roots. Such changes have been described in detail for various crops. They include traits that increase: (a) the solubilisation of inorganic P and the breakdown of organic P compounds in the soil, (b) the volume of soil explored by roots or the root surface area available to capture soluble P, (c) the rate of P uptake across the plasma membrane of root cells, and (d) whole-plant traits that affect root growth and nutrient capture [6, 7]. “Topsoil foraging” is believed to be the most efficient root ideotype for P acquisition [5, 8] presuming that P is concentrated in the top layers of the soil. The most favourable root system architecture (RSA) for P acquisition is a combination of i) large root biomass, root / shoot biomass ratio, cortical aerenchyma, root surface area in the topsoil and in patches of high P phytoavailability, ii) ability to form mycorrhizal symbioses, acidify the rhizosphere, release organic compounds and phosphatases, and iii) greater phosphate (P_i_) uptake capacity of root cells [5, 8]. In this context, a greater number of lateral roots was found in *Brassica oleracea* L. accessions with average to high yields at suboptimal P supply and the number of lateral roots increased with yield potential [9]. Furthermore, total lateral root length and lateral root growth rate were larger for accessions that had greater yields at small and large P supply, while root angle did not appear to be related to greater yields [9]. Similar adaptations have been observed for other crops, e.g. soybean (*Glycine max* (L.) Merr.) [10] and oilseed rape (*Brassica napus* L.) [11]. In *Arabidopsis thaliana* (L.) Heynh., low P availability promotes the development of a highly branched root system to the detriment of the primary root, characterised by greater production of lateral roots and root hairs [12, 13].

The alteration of root architecture (i.e. the spatial configuration of a root system) is a powerful vehicle for the development of crop plants with efficient P_i_ acquisition [7, 14, 15]. Nevertheless, root exudates (i.e. organic metabolites and inorganic species released by the roots to their surroundings) play a complementary role in the mobilisation of sparingly soluble mineral elements in the rhizosphere [16]. Root exudation has been shown to be influenced strongly by the nutritional status of the plant and both the quantity and the composition of exudates differ depending on the particular nutrient (P, Fe, K or N) deficiency [17]. Efficient soybean genotypes, for example, respond to low P availability by exuding a greater quantity of organic compounds into the rhizosphere, which increases P availability and results in the crop meeting P requirements, producing consistent biomass and seed yield with reduced fertiliser addition [18]. Furthermore, the (bio) availability of P_i_ can affect the overall profile of the root exudates [19]. In P-deficient strawberry (*Fragaria × ananassa* cv. Elsanta) plants, for example, the concentrations of citric acid, galactaric acid, malic acid, lysine, proline, and sorbitol-6-phosphate in root exudates were larger in P-deficient plants than in P-replete plants [20].

Phosphorus-deficiency in plants can also affect the uptake of other essential and non-essential mineral elements [19, 21]. In particular, P deficiency can induce zinc (Zn) over-accumulation [22–25]. This interaction has been attributed to (a) biochemical changes affecting cell membranes [26] and/or increased apoplastic uptake of Zn [27], (b) morphological changes in RSA that allow greater volumes of soil to be exploited for Zn acquisition [5], or (c) cross talk of molecular signalling networks, by which P_i_ deficiency upregulates the expression of genes encoding Zn transporters [22, 28, 29]. Conversely, P-induced Zn deficiency has been reported for some crops [30–34]. Since Zn deficiency in plants is linked with Zn malnutrition in animals, understanding Zn nutrition in plants in relation to environmental factors is an important subject [21, 23, 26, 35–37].

As in leaves [38], the cell-type specific distribution of elements in roots depends on the phylogenetic placement of the plant species and on the environment. Although P nutrition has been studied in a wide range of plant species, particularly in crops, studies of the tissue-specific distribution of P in roots are rare and typically a serendipitous result of using multi-element analytical techniques. In general, a progressive increase in cellular P from the rhizodermis towards the endodermis, where the majority of P is located, and smaller P concentrations in cells located within the stele has been reported [39–42].

The aim of this study was to determine root traits (root exudates, RSA, gene expression and tissue-specific allocation of P in roots) that (a) play a role in P-use efficiency and (b) contribute to larger shoot Zn concentration using two contrasting *B. oleracea* accessions.

## Results

### Hydroponic experiments

**Fig. 1:**
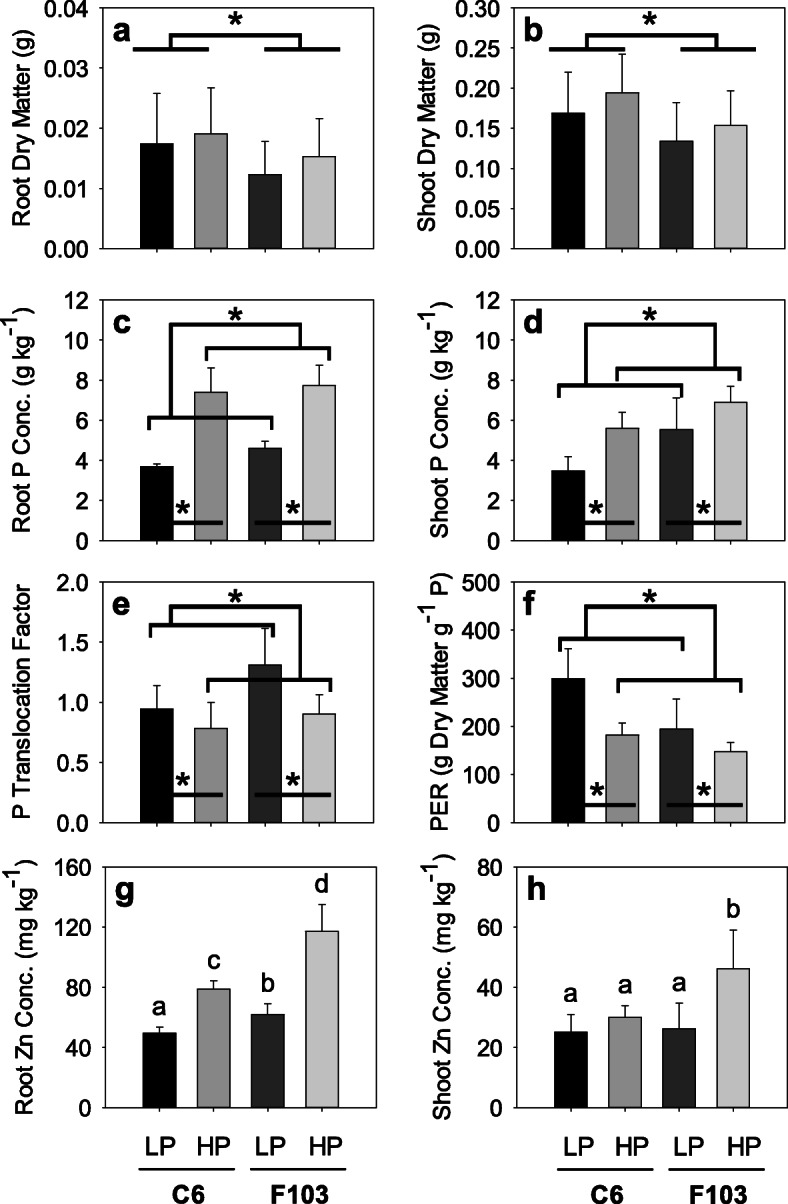
Root and shoot dry matter (**a**, **b**), root and shoot phosphorus (P) concentration in dry matter (**c**, **d**), P translocation factor (shoot P/root P; **e**), P efficiency ratio (PER; **f**), a measure of P use efficiency calculated as Shoot Dry Matter/(Shoot P Concentration × Shoot Dry Matter), and root and shoot zinc (Zn) concentration in dry matter (**g**, **h**) of two *Brassica oleracea* accessions (C6 and F103) grown hydroponically for two weeks in solutions containing 0.025 mM P (*Low P*; LP) and 0.25 mM P (*High P;* HP). Data are means (*n* = 18 for dry weight and *n* = 5–6 for concentrations, translocation factor and PER) and standard errors. Asterisks and different letters indicate statistically significant differences (two-way analysis of variance followed by Holm-Sidak post hoc test at *P* < 0.05)

Both *B. oleracea* accessions grew well at both *Low P* and *High P* supply (henceforth referred to as treatments) and showed no P_i_ deficiency symptoms (Additional file 1: Fig. S1). There was no significant effect (Two-way ANOVA) of the treatments on the root lengths and root and shoot dry matter, but a statistically significant genotype effect was found (Additional file 2: Table S1), with the F103 accession having longer roots (28.2 ± 0.86 cm) than the C6 accession (25.6 ± 0.88 cm), and the C6 accession having larger average root and shoot dry matter than the F103 accession (Fig. 1a, b).

There was a statistically significant effect of genotype and treatment on root and shoot P concentrations, P translocation factor (shoot P / root P concentration) and P efficiency ratio (PER) (Additional file 2: Table S1). The largest root P concentrations were measured in plants receiving *High P* supply, and the shoot P concentration of the F103 accession receiving a *Low P* supply did not differ from the shoot P concentration of the C6 accession receiving a *High P* supply (Fig. 1c, d). Phosphorus translocation factors were larger in the F103 accession than in the C6 accession in both treatments (Fig. 1e). The greatest PER was found in the C6 accession receiving a *Low P* supply and the smallest in the F103 accession receiving a *High P* supply (Fig. 1f). The root, shoot and plant P contents were significantly larger in both accessions in the *High P* supply than in the *Low P* supply (Additional file 1: Fig. S2).

There was a statistically significant effect of genotype, treatment and their interaction on both root and shoot Zn concentrations (Additional file 2: Table S1). The largest root and shoot Zn concentrations were measured in the F103 accession receiving a *High P* supply (Fig. 1g, h). Zinc translocation factors did not differ between the two accessions but were larger in the *Low P* supply (on average 0.49 ± 0.12) than in the *High P* supply (on average 0.39 ± 0.08).

Two-way ANOVA of shoot and root S, K, Ca, Mn and Fe concentrations revealed that i) there were no significant effects of genotype, treatment or their interaction on shoot Ca, S, K, Mn or Fe concentrations, ii) root Ca, K and Mn concentrations were affected by genotype, iii) *High P* supply resulted in larger root Ca, S, K, Mn and Fe concentrations than *Low P* treatment, and iv) there were no genotype x treatment interactions on root S, K, Ca, Mn or Fe concentrations (Fig. S3).

### Metabolome profiling of root exudates

**Fig. 2:**
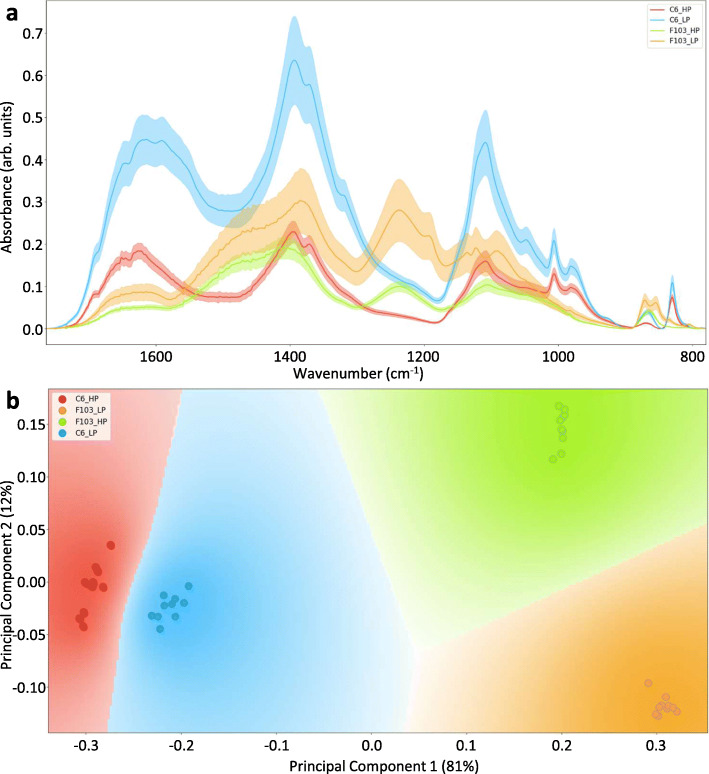
Attenuated total reflection-Fourier transform infrared analysis of root exudates collected from two *Brassica oleracea* accessions (C6 and F103) grown hydroponically for two weeks in solutions containing 0.025 mM phosphorus (P; *Low P*; LP) and 0.25 mM P (*High P;* HP). Average spectra of ten measurements ± standard deviation (**a**) and principal component analysis, which depicts the first two principal components for response variables grouped by treatments and accessions (**b**)

The comparison of the ATR-FTIR spectra of the root exudates revealed large differences between the accessions and the treatments (Fig. 2). In the characteristic range of the spectra (wavenumber 800–1700 cm^− 1^) the functional group profiles of the two accessions differed clearly (Fig. 2a). Considering the whole characteristic range as the source of variation, the PCA showed separation of the two accessions, mainly in the principal component one (PC1), which explained 81% of the variation (Fig. 2b). In the principal component two (PC2), which explained 12% of the variation, strong separation between treatments was seen for the F103 accession only. Bands contributing distinctly to the separation in the dimension of PC1 and as such to the separation of the accessions were those between ≈1200–1300 cm^− 1^, between ≈1400–1550 cm^− 1^ and between ≈1620–1690 cm^− 1^ (Additional file 1: Fig. S4). Several other minor contributions were also apparent, e.g. ≈830 cm^− 1^, ≈870 cm^− 1^, ≈890 cm^− 1^, ≈970 cm^− 1^, ≈1100 cm^− 1^, ≈1110 cm^− 1^, ≈1380 cm^− 1^ and ≈1400 cm^− 1^ (Additional file 1: Fig. S4). Because bands in ATR-FTIR spectra are a combination of symmetric and asymmetric stretching, bending and wagging vibrations of compounds present in the samples the attribution of a band at a certain wavelength to a particular compound is complicated [43, 44]. However, some general observations can be made in conjunction with published data ([45] and references therein). For example, bands between ≈1620–1690 cm^− 1^ are mainly a result of vibrations from protein (amide I) and pectin, the bands between ≈1400–1550 cm^− 1^ are mainly a result of vibrations of carboxyl groups from lignin, pectin, various polysaccharides and protein (amide II), while bands between ≈1120–1300 cm^− 1^ are mainly a result of vibrations of lignin, proteins (amide III), nucleic acids and various polysaccharides [45]. Thus, it appears that the largest contributors to the separation of the two accessions were proteins. By contrast, bands contributing distinctly to the separation in the dimension of PC2 and as such to the separation of the *Low P* and the *High P* treatments in the F103 accession were those between ≈1560–1700 cm^− 1^, between ≈1390–1560 cm^− 1^, between ≈1120–1380 cm^− 1^, between ≈960–1090 cm^− 1^ and at ≈880 cm^− 1^ and at ≈830 cm^− 1^ (Additional file 1: Fig. S5). These contributing bands can be assigned to the vibrations of pectin and various polysaccharides (e.g. ≈1020–800 cm^− 1^, ≈1155 cm^− 1^, ≈1260–1300 cm^− 1^ and ≈1370 cm^− 1^, ≈1400–1460 cm^− 1^ [45], which implies that the largest differences in root exudates in plants of the F103 accession with *Low P* and *High P* supply may be due to the presence of polysaccharides.

**Fig. 3:**
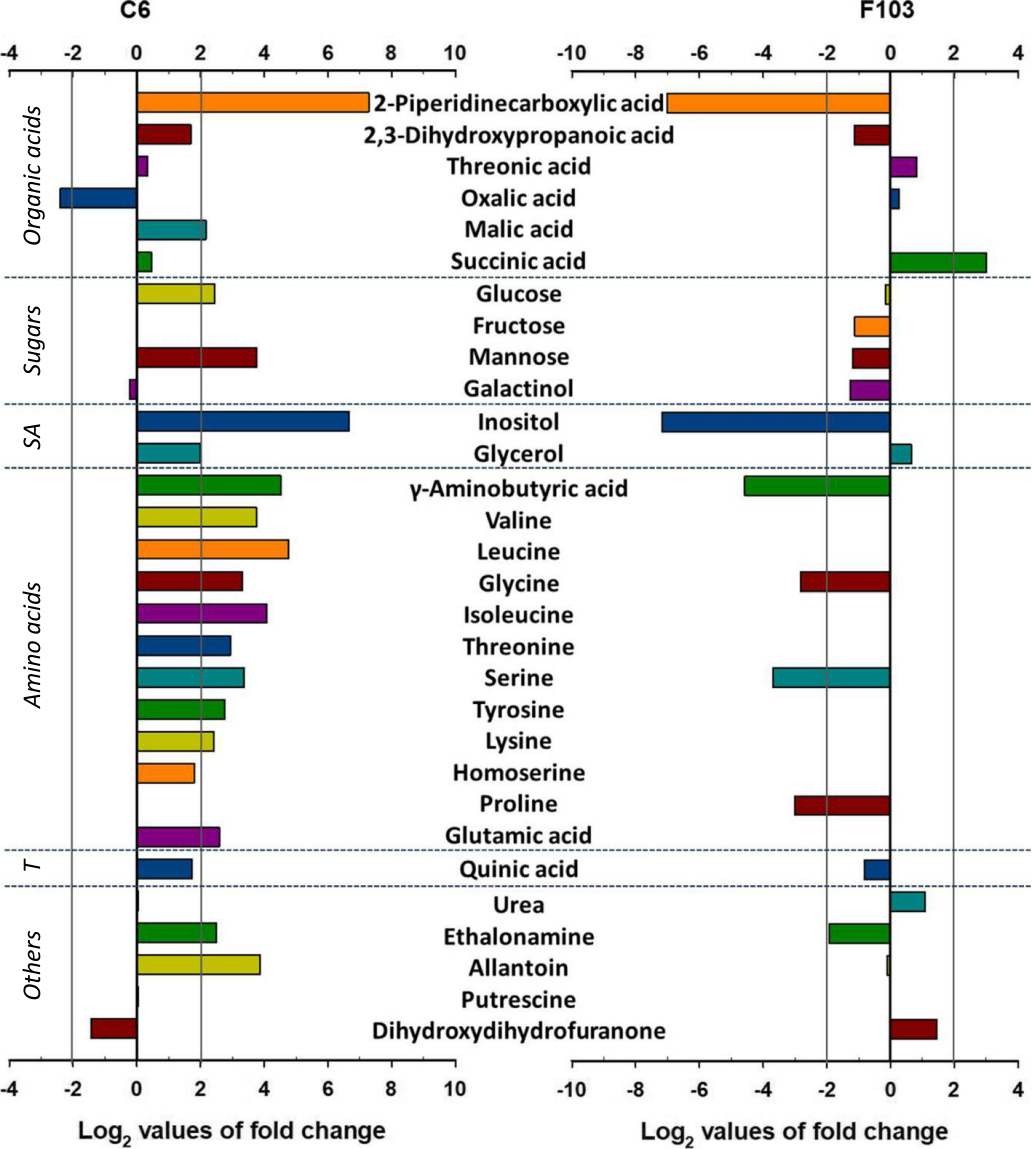
Log2 values of fold change (*Low phosphorus (P)* / *High P*) in relative concentrations of metabolites in root exudates detected in two *Brassica oleracea* accessions (C6 and F103) grown hydroponically for two weeks in solutions containing 0.025 mM P (*Low P*) and 0.25 mM P (*High P*). SA, sugar alcohols; T, tannins

Using GC-MS, a total of 39 polar compounds (organic acids, sugars, sugar alcohols, amino acids, lignins and others) were identified in root exudates of the C6 accession and 34 compounds in root exudates of the F103 accession (Additional file 2: Table S2). In all instances, the relative concentrations of metabolites were larger in root exudates of the C6 accession than the F103 accession. In root exudates from the C6 accession three compounds (oxalic acid, galactinol and dihydroxydihydrofuranone) were found to be more abundant in the *High P* treatment than in the *Low P* treatment with one compound (galactose) detected in the *High P* treatment only and ten compounds (fumaric acid, fructose, sucrose, galactosyl glycerol, phenylalanine, oxoproline, tryptophan, ß-alanine, proline and methionine) detected in the *Low P* treatment only (Additional file 2: Table S2). The remaining 25 compounds were more abundant in the *Low P* treatment compared to the *High P* treatment in root exudate from the C6 accession. By contrast, in root exudate from the F103 accession fourteen compounds were more abundant in the *High P* treatment than the *Low P* treatment and thirteen compounds were detected in the *High P* treatment only and one compound (fumaric acid) was detected in the *Low P* treatment only (Additional file 2: Table S2). The remaining six compounds were more abundant in root exudates from the *Low P* treatment compared to the *High P* treatment. Log2 fold changes (comparing *Low P* with *High P* treatments) are shown in Fig. 3. The fold changes were calculated for the 30 compounds detected in both treatments. Significant changes in exudation (Log2 fold change > 2) were found for eighteen compounds in the C6 accession and seven in the F103 accession.

### Root architecture

**Fig. 4:**
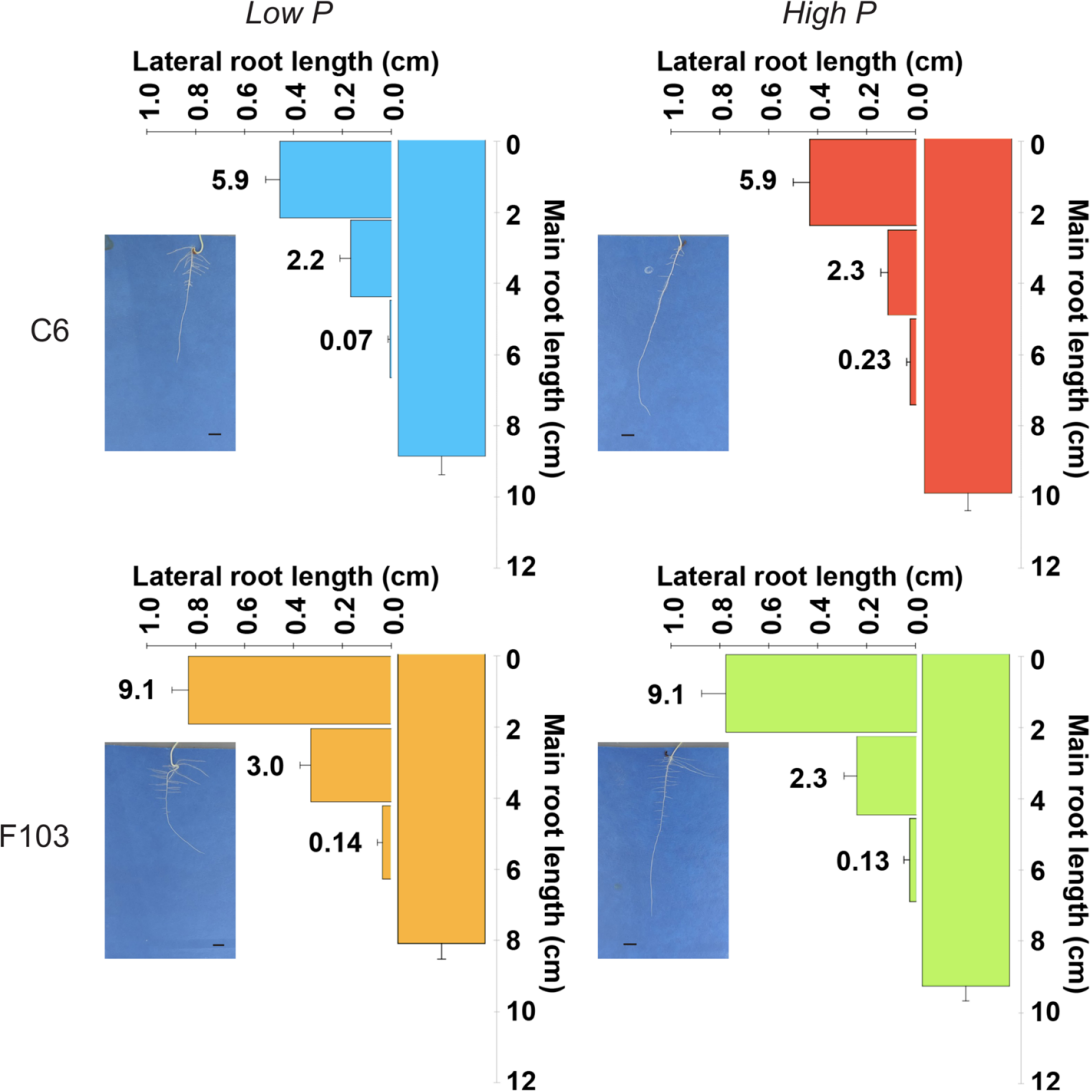
Photographs of root system architecture and average main and lateral root length and number of lateral roots in two *Brassica oleracea* accessions (C6 and F103) grown in a high throughput root phenotyping system for two weeks and supplied with solutions containing 0.025 mM P (*Low P*) and 0.25 mM P (*High P*). Length of lateral roots was determined in each quartile of the main root length, hence the three horizontal columns (in the fourth quartile there were no lateral roots). Numbers to the left of the columns indicate the average number of lateral roots per quartile. Data are means and standard errors. Scale bars indicate 1 cm

There was an apparent difference in root system architecture (RSA) between the two accessions, particularly in the occurrence and the length of the lateral roots in the uppermost part of the main root (close to the shoot; representative photographs of RSA of the accessions grown in different treatments are shown in Fig. 4). For this reason, the number and length of lateral roots were determined in each of four equidistant parts along the main root axis (first, second, third and fourth quartiles were distinguished with the first quartile positioned at the top of the main root). The length of the main root was affected by treatment and not by genotype or the interaction between P treatments and genotype (Two-way ANOVA; Additional file 2: Table S3). The average main root length was 8.5 cm in the *Low P* treatment and it increased to 9.6 cm in the *High P* treatment. Data for lateral root length and lateral root number was not normally distributed, therefore ANOVA on Ranks was performed. The analysis revealed significant differences in lateral root lengths between the accessions in the *Low P* treatment, but not in the *High P* treatment. In addition, there was no statistically significant difference in the lateral root length between the two treatments for either the C6 or the F103 accession. The C6 accession had fewer lateral roots that were shorter than those of the F103 accession in the uppermost quartile. This trend was still seen in the second quartile, although the differences were not as large (Fig. 4). In the third quartile lateral roots were only occasionally found and their occurrence was not affected by any of the variables (Additional file 2: Table S3). In the fourth quartile lateral root emergence had not yet occurred and thus lateral roots were not visible.

### Distribution of phosphorus in the root cross sections

**Fig. 5:**
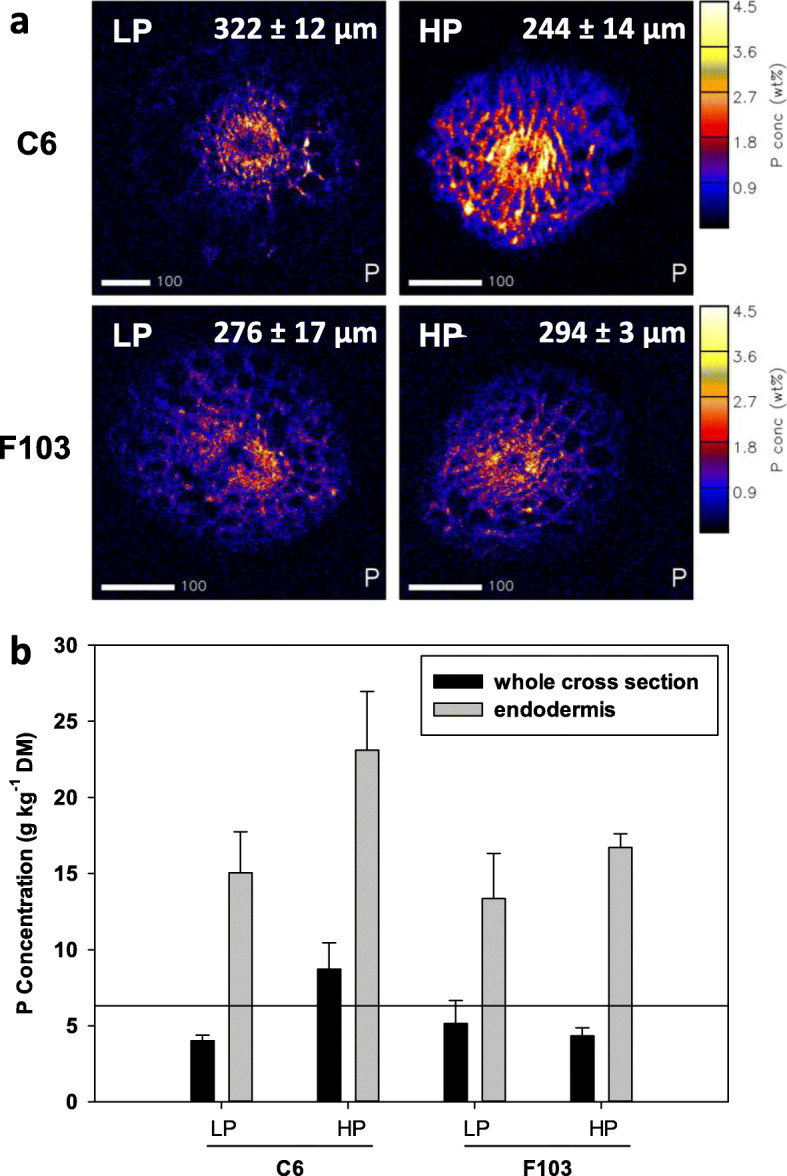
Representative quantitative phosphorus (P) distribution maps in root cross sections of two *Brassica oleracea* accessions (C6 and F103; a) grown in a high throughput root phenotyping system for two weeks and supplied with solutions containing 0.025 mM P (*Low P;* LP) and 0.25 mM P (*High P*; LP). Values in the top right corners of P distribution maps indicate mean diameter of root cross sections (*n* = 3–8). Average P concentration in dry matter of whole root cross sections and endodermis (*n* = 3–7; b). Reference line in panel b indicates average whole-cross section concentration (*n* = 18). Shown are means ± standard errors

Concentrations of elements in particular tissues differ along the length of plant roots [46–49]. To be able to compare the distributions of P in the two treatments and the two accessions quantitatively, regions of the roots of similar developmental stage were examined. Because the shortest distance from the root tip and the first lateral root was determined to be at 2.6 cm in the F103 accession, all cross sections were taken between 2.0 and 2.5 cm above the root tip (the maturation zone). Resulting cross sections were on average 288 ± 9 μm in diameter. The root cross sections were larger in the C6 accession than in the F103 accession receiving *Low P* supply, while the opposite was observed in the *High P* supply (Fig. 5a).

Whole root-cross sections contained on average 6.30 ± 0.90 g P kg^− 1^ (Fig. 5b). The largest concentrations of P in root cross sections of both *B. oleracea* accessions were found in the endodermis (Fig. 5b), a layer of cells surrounding the vascular bundle. Although accession and treatment effects on the distribution of P within the root were not significant, some observations can be made. On average less P was measured in whole cross-sections and in the endodermis of roots of the C6 accession receiving the *Low P* treatment compared to the *High P* treatment, whilst such trends were not observed for roots of the F103 accession (Fig. 5b). In the *High P* supply, the C6 accession had a larger P concentration in the endodermis than the F103 accession.

### Gene expression

**Fig. 6:**
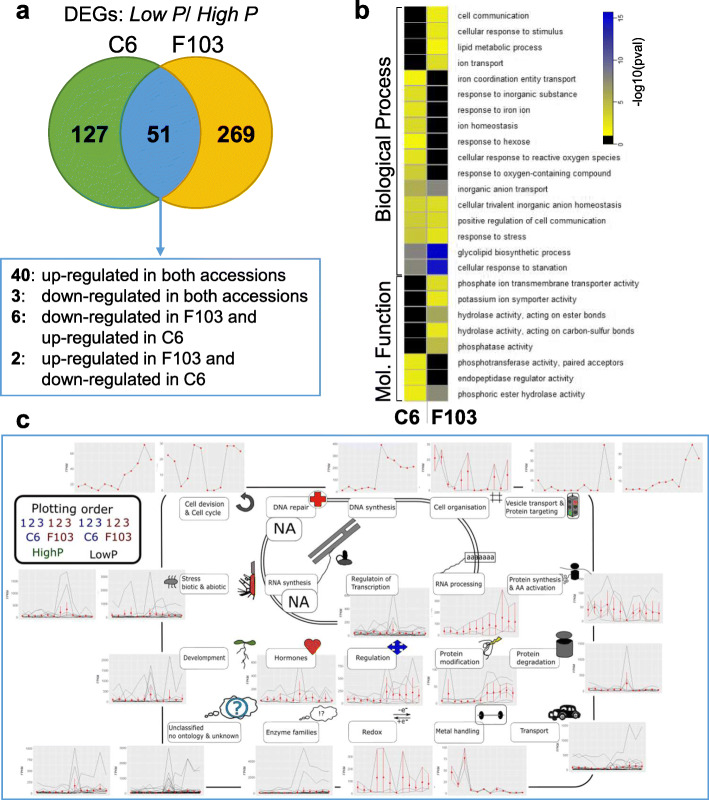
Comparative analysis of differentially expressed genes (DEGs) in roots of two *Brassica oleracea* accessions (C6 and F103) grown hydroponically for two weeks in solutions containing 0.025 mM P (*Low P*) and 0.25 mM P (*High P*). Differentially expressed genes were defined by comparing the gene expression in the *Low P* treatment with the gene expression in the *High P* treatment for each of the *B. oleracea* accession. **a** Schematic representation of the common and accession-specific DEGs. **b** Gene ontology analysis of enriched functional categories of these DEGs. Depicted are *p*-values to highlight the significance of the over-representation of the corresponding functional category. Only child terms are displayed here. The full analysis can be found in Additional file 2: Table S6. **c** A MapMan analysis illustrating responses of functional pathways to the *Low P* treatment. The image was designed after the MapMan output for cellular overview. Line graphs below categories show expression of respective genes in response to decreasing P supply with the average and the confidence interval are shown in red. *Arabidopsis thaliana* orthologues of *B. oleracea* P-responsive DEGs were used as input together with expression values detected in the two *B. oleracea* accessions

RNAseq data shown here originate from a previously published dataset [29] and were processes anew by concentrating on the different P supply (*Low P* and *High P*) only; differences in gene expression in response to Low P supply were assessed. In the C6 accession 178 genes were differentially regulated in *Low P* supply in comparison to *High P* supply while in the F103 accession 320 DEGs were detected (Fig. 6a). These DEGs (Additional file 2: Table S4) are referred to as P-responsive. The two accessions had 51 P-responsive DEGs in common, of which the majority (40) were up-regulated in the *Low P* treatment. An example of these up-regulated DEGs is Bo9g181910, which is annotated as a phosphate starvation response gene. Only three DEGs were down-regulated in both accessions as a response to *Low P* supply. Among them the Bo5g008780 is annotated as the high affinity nitrate transporter 2.1. Eight DEGs showed contrasting responses to P supply in the two accessions. Only one P-responsive gene linked with Zn transport was found (Bo1g021930) and was down-regulated in the C6 accession in the *Low P* treatment. We searched for over or underrepresented gene ontology (GO) terms among the P-responsive DEGs by comparing them statistically to the respective GO group size in all expressed *B. oleracea* genes (Fig. 6b). All GO enrichments can be found in Additional file 2: Table S5. We detected GO term enrichments for both accessions, but only rarely were these enrichments common to both accessions. One of the common enrichments in GO terms was “glycolipid biosynthetic process” suggesting that both accessions respond to *Low P* supply by changing their lipid metabolism. A MapMan analysis of the gene expression changes, annotated with their *A. thaliana* orthologues, illustrates which cellular processes are affected schematically (Fig. 6c). The expression profiles of P-responsive DEGs (depicted as their orthologues in *A. thaliana* listed in the Additional file 2: Table S6), are shown in the line graphs below each group. Processes strongly impacted by P starvation include those influencing the regulation of transcription, RNA processing, protein synthesis and modification, responses to stress, redox activities, cell signalling, hormonal regulation, metal homeostasis and solute transport.

## Discussion

As expected, when grown for 2 weeks in hydroponics the two selected *B. oleracea* accessions exhibited measurable ionomic (Fig. 1, Additional file 1: Figs. S2,3), metabolic (Figs. 2, 3), genetic (Fig. 6), and morphological (Figs. 4, 5) responses to reduced P availability. Both accessions up-regulated the expression of a gene associated with responses to P-deficiency (Bo9g181910), which suggests that the *Low P* treatment caused sub-optimal plant P nutrition. However, the responses to reduced P availability occurred before visible deficiency symptoms in shoots were observed, such as a reduction in shoot growth (Fig. 1b) and the accumulation of purple anthocyanin pigmentation (Additional file 1: Fig. S1), which is indicative of systemic P deficiency [50]. In addition, in both accessions an adequate shoot P concentration was measured in plants receiving either a *High P* or a *Low P* supply (Fig. 1d). Nevertheless, the two accessions appeared to achieve P sufficiency [51] by employing different mechanisms. The C6 accession relied more on changes in the quantity and composition of root exudates, while the F103 accession relied more on an extensive root system which, surprisingly, did not depend on the P supply. These conclusions were based on the following observations i) in hydroponics (a) the C6 accession had larger biomass (Fig. 1b) but smaller shoot P (Fig. 1d) and Zn (Fig. 1h) concentrations, and larger PER, a measure of P-use efficiency [9], especially when receiving *Low P* supply, than the F103 accession (Fig. 1f), (b) the C6 accession exuded a larger number of polar compounds and their relative concentrations were larger than in the F103 accession (Fig. 3 and Additional file 2: Table S2), and (c) roots of the C6 accession had fewer DEGs than roots of the F103 accession, and ii) in the HTRP experiment, the F103 accession had a larger number of lateral roots, which were longer than in the C6 accession (Fig. 4). It appears that in the F103 accession growing roots was more energy demanding than was the root exudation in the C6 accessions, in agreement with recent estimates [16, 52].

Root exudates are employed by many plants to release bioavailable nutrients from the soil and to interact with rhizosphere microbiota and herbivores [17, 53, 54]. Their mode of action, in relation to plant nutrition, can include direct effects on nutrient solubility, as exemplified by the effects of organic acids released into the rhizosphere, or indirect effects, such as the attraction of rhizosphere microorganisms that increase the bioavailability of nutrients in the soil [17]. Although the quantity and the composition of root exudates have been shown to be species and genotype-specific [55, 56], the differences in the root exudate composition between the two *B. oleracea* accessions studied here, captured by ATR-FTIR (Fig. 2), was still remarkable. These differences were apparent despite potential issues [57, 58] with the length of root exudate collection, which exceeded the proposed optimal time for washing [59]. Since both accessions were exposed to the same procedure, the differences observed in their root exudate composition and its response to P supply can be attributed to genotypic and treatment effects. The significant response to the *Low P* supply observed in the C6 accession, suggests a strong interaction between root exudates and P-stress in this accession. Proteins appeared to contribute most prominently to the differences in exudates between the two accessions. Indeed, the number, concentration and types of amino acids (Fig. 3 and Additional file 2: Table S3) differed significantly between the two accessions as was revealed by untargeted profiling of polar root exudates using GC-MS. Amino acids in root exudates have, beside sugars and sugar alcohols, been reported to tailor microbial communities, because they represent carbon and nitrogen substrates for microbial growth [60–62]. Previously, P deficiency was shown to stimulate the release of γ-aminobutyric acid and carbohydrates in maize grown hydroponically [17], as was observed for the C6 accession. However, the concentrations of these compounds in exudates from roots of the F103 accession were reduced under P deficiency.

Exudation of organic acids by roots has been linked to P mobilisation in the soil [61], although the magnitude of the effect is debated [63] and there are large differences in the quantity and composition of organic acids released from roots among species and genotypes [16, 64]. In cabbage grown hydroponically, the exudation of citrate, malate and succinate was induced by P deficiency [65], as it was in canola grown in the soil [63], but not when canola was grown in sand culture supplied with different P sources [66]. The P mobilising ability of low-molecular-weight organic acids follows the order: citric > oxalic > tartaric > malic acid [67]. Citric acid, the organic acid that most often increases in concentration in root exudates in response to P-deficiency [68] was not detected in our study. Instead, malic acid, in the C6 accession, and succinic acid, in the F103 accession, appeared to increase most in concentration in response to reduced P availability. The largest change in concentration in both accessions, although in opposite directions, was of 2-piperidinecarboxylic acid, whose role has not been studied in detail in root exudates but has been found to increase in *A. thaliana* roots when grown in alkaline solution [69]. Alkaline soil is known to reduce the availability of nutrients such as P [70]. It is possible that a compound like 2-piperidinecarboxylic acid that might mobilise P in alkaline conditions might also contribute to P mobilising when P availability is restricted by a *Low P* supply caused by inadequate total P concentrations in the soil.

Lateral roots play an important role in P acquisition by increasing soil exploration and the surface area of the root system for P uptake [14]. A greater number and length of lateral roots have been shown to contribute significantly to higher yields regardless of the external P concentrations in genotypes with high P uptake efficiency [9]. The observed RSA with numerous long lateral roots closer to the upper parts of roots (first quartile) in the F103 accession (Fig. 4) matches the “topsoil foraging” phenotype, which is believed to be the most efficient root ideotype for efficient P acquisition [5, 8]. In this accession, however, although relatively large shoot P concentrations were measured, this was not associated with a corresponding large biomass accumulation, which indicates poor physiological P-use efficiency [9]. In the HTRP experiment the largest concentrations of P in root cross sections was found in the endodermis (Fig. 5) in agreement with previous reports in various plant species [39, 41, 42, 71]. The endodermis represents a key apoplastic barrier to the radial transport of water and ions to the vascular system of the plant and as such it acts as a checkpoint in nutrient delivery to the shoot [27]. The observation that the C6 accession accumulated slightly more P in the endodermis at the *High P* supply than the F103 accession, could not be correlated with any of the traits studied, including differences in the P translocation factor. Further studies will be required to determine whether there is any link between P accumulation in the endodermis and a physiological function.

Despite accession C6 having fewer, and shorter, lateral roots than the F103 accession, the root cross section diameter was larger in the C6 accessions compared to the F103 accession receiving *Low P* supply (Fig. 5a). The size of the root cross sections was investigated because common bean (*Phaseolus vulgaris* L.) genotypes with reduced root secondary growth, which caused smaller root cross sectional areas, smaller root respiration, and longer roots than other genotypes) produced significantly larger shoot biomass and contained larger shoot P concentrations than other genotypes when grown with a low P supply [72]. However, PERs were not significantly different between common bean genotypes with reduced or advanced secondary growth of roots [72]. In the study described here the largest root diameter (Fig. 5) coincided with the largest PER (Fig. 1f), which occurred in the C6 accession receiving the *Low P* supply. It may be that other root traits, such as biochemical traits, which were not studied by [72], contributed to the greater PER. Indeed, in response to limiting soil P, species with thicker roots relied predominantly on higher solonisation by AMF to compensate for a low root absorptive surface and/or more P-mobilizing exudates to mine sparingly soluble P in the rhizosheath [73].

The large differences in metabolic and morphological root traits studied in the two accessions were supported by extensive differences in the DEGs in roots of the two accessions illustrated both as GO enrichments and MapMan analysis (Fig. 6). More DEGs in roots were accession-specific than were common to both accessions. The F103 accession responded to the *Low P* supply with more DEGs in roots than the C6 accession. Even when the GO enrichment was found to be common to both accessions, namely “glycolipid biosynthetic process” and “cellular response to starvation”, the responses in the F103 accession were more significant. Lipids play crucial roles in the ability of plants to acclimate to, and survive, P scarcity [74–76], because some phospholipids can be replaced with non-phosphorus glycolipids and sulfolipids [77]. In general, the majority of GO processes were accession-specific, an observation which was further supported by the MapMan analysis of the gene expression of *B. oleracea*, annotated with their *A. thaliana* orthologues (Fig. 6c). The cell function overview showed repression for categories such as “metal handling” in the C6 accession with *Low P* supply. In this category, expression of genes linked with Fe homeostasis (such as Ferric Reduction Oxidase 2 (FRO2), Ferric Reductase Defective (FRD3) and Nicotianamine Synthase 2 (NAS2) [78]) were strongly down-regulated in the C6 accession in the *Low P* treatment. However, these expression changes were not accompanied by significant differences in root or shoot Fe concentrations (Additional file 1: Fig. S3). These observations are in contrast to previous observations in *A. thaliana* in which Fe acquisition was greater in P-deficient plants than in P-sufficient plants and the induction of different Fe homeostasis genes, such as NAS3 and FER1, were correlated with P-depletion [79]. The difference in response of *B. oleracea* and *A. thaliana* might be related to the Fe concentration in roots of *B. oleracea* accessions grown in the *Low P* treatment being smaller than when grown in the *High P* treatment (Additional file 1: Fig. S3), while the opposite was observed in *A. thaliana*. It is also possible that the response studied here is species specific, highly dynamic, depends on the level of P-deprivation stress or is related to other environmental factors, such as medium composition or the amount of available Fe. In addition, *A. thaliana* has been shown to have a very specific Fe-response to low P supply. For example, in *A. thaliana* an Fe-toxicity related reduction of main root elongation occurs at low P supply, which is uncommon in other plant species [80]. The expression of genes associated with “transport” processes, such as those encoding the H^+^-exporting ATPase (AHA2), Nitrate Transporter 2;1 (NRT2;1), Phosphate Transporter 1;4 (PHT1;4), Phosphate Transporter 3 (PHT3), Iron-Regulated Transporter 1 (IRT1) and a High Affinity potassium transporter (HAK5), responded in a complex manner to contrasting P supply, perhaps reflecting the network of interactions between elements in plants [81]. These changes might allow interesting insights to elemental homeostasis at low P supply. Overall, our analysis of gene expression highlights the distinct responses of the C6 and the F103 accessions to P deprivation as well as their common response of altering lipid biosynthesis. In addition, both C6 and F103 appear to utilise ROS signalling pathways to induce responses to *Low P* supply and induce phosphotransferases and phosphatases.

Interactions between P and Zn were also evaluated, since a negative relationship between increasing P supply and shoot Zn concentration was expected based on observations in several crops [23, 31, 33, 82] and because the shoot concentration of no other element measured responded to genotype, treatment and their interaction (Additional file 1: Fig. S3). In addition, low P supply has been reported to result in shoot Zn-overaccumulation [22, 24, 25]. In efforts to increase shoot Zn concentration in edible crops to improve human Zn nutrition [83, 84], the first interaction is undesired and therefore crops or varieties with the ability to circumvent it are being sought. Although there was a clear response in root Zn concentrations with increasing P supply in both accession (Fig. 1g), only in the shoot of the F103 accession was Zn concentration increased in response to *High P* supply (Fig. 1h). This observation differs from previous observation in the majority of *B. oleracea* genotypes [23] and mustard (*Brassica juncea* (L.) Czern.) [32, 82] in which shoot Zn concentrations were reduced by increasing P supply. Indeed, shoot Zn concentrations in both the C6 and F103 accessions were reduced when plants were grown in peat-based compost with increasing P supply [23].

Since there was no significant response in the number of lateral roots or their length in the two accessions in any of the root quartiles, it appears that root architecture did not play a role in their contrasting Zn acquisition. However, lateral root density of field-grown oilseed rape, but not primary root length and lateral root length, was significantly positively related to leaf Zn concentrations [85]. Larger shoot Zn concentrations in the F103 accession than in the C6 accession were also reported by [23] (Additional file 2: Table S7), which suggests these two accessions differ in their ability to accumulate Zn. Indeed, greater expression of a gene encoding a putative Zn-transporter in roots of the F103 accession than in roots of the C6 accession was observed when plants were grown in an experiment with the same design with sufficient Zn supply [29]. Since Zn translocation factors did not differ between the two accessions, although there was a decrease in translocation of Zn when plants received *High P* supply compared to the *Low P* supply, it appears that the Zn uptake into roots played a significant role in achieving higher Zn concentration in shoots.

## Conclusions

The results obtained from the experiments performed here demonstrate extensive within-species differences in the responses of the ionome, root exudate composition and gene expression to the two P treatments studied. They provide a basis for experiments in mini-rhizotron systems that enable non-destructive root imaging and analysis [11, 86], which will provide validation of the results in a more realistic environment.

## Methods

### Plant material

Two *B. oleracea* accessions: C6 (“Winterbor F1” kale, *B. oleracea* var. *sabellica*) and F103 (broccoli, *B. oleracea* var. *italica*) with contrasting P use efficiencies (Additional file 2: Table S7) were investigated. The choice of these two accessions was made by stepwise selection. First, eight accessions with a contrasting P use efficiency measure, namely the P efficiency ratio (PER) calculated as Yield_low P_/(P_low P_ × Yield_low P_) or Yield_high P_/(P_high P_ × Yield_high P_), were selected from a diversity foundation set of 376 genotypes of *B. oleracea* [9, 23]. Second, these eight accessions were grown hydroponically for 2 weeks with different combinations of P and Zn supply and two accessions, F103 and C6, were selected based on the contrasting responses of their ionomes to these treatments [29]. Seeds of the F103 accession were obtained from the Genetic Resource Unit, Warwick Crop Centre of the University of Warwick (where this accession is kept under the HRIGRU04701 number; https://warwick.ac.uk/fac/sci/lifesci/wcc/gru/). Seeds of the C6 accession were commercially available from Sakata Seed America Inc. (https://www.sakata.com/) as a Winderbore F1 genotype with the common name of Borecole. The accessions were first grown in a glasshouse for seeds (seed bulking). For the hydroponic experiments, these seeds were germinated on the surface of filter paper moistened with deionised water in a Petri dish placed at 16 °C in the dark for 5 days. For high throughput root phenotyping (HTPR) experiments, seeds were used directly, without pre-germination, and were placed onto blue germination paper.

### Hydroponic experiments

The two *B. oleracea* accessions were grown at two different P concentrations: 0.025 mM P (*Low P*) and 0.25 mM P (*High P*). The *High P* nutrient solution contained 2 mM Ca (NO_3_)_2_, 2 mM NH_4_NO_3_, 0.75 mM MgSO_4_, 0.5 mM KOH, 0.25 mM KH_2_PO_4_, 0.1 mM FeNaEDTA, 30 μM H_3_BO_3_, 25 μM CaCl_2_, 10 μM MnSO_4_,3 μM CuSO_4_,1 μM ZnSO_4_ and 0.5 μM Na_2_MoO_4_. The *Low P* nutrient solution had a similar composition, except that it contained 0.025 mM KH_2_PO_4_ and 0.225 mM KCl to provide an equivalent K supply. The experiment was performed as described in detail previously [29]. There were four buckets (24 plants) for each accession and each treatment.

### Gene expression study through RNAseq analysis

Procedures for RNA extraction from roots (*n* = 3), library preparation, sequencing and bioinformatics analysis have been described in detail previously [29]; this previously published RNAseq dataset was processed anew by concentrating solely on the two P treatments: the *Low P* and the *High P* treatments. Differentially expressed genes (DEGs) were calculated as a ratio of the gene expression in the *Low P* treatment in comparison to the gene expression in the *High P* treatment. A filter of fold change > 2 and a diverge probability greater than or equal to 80% was applied to define the P-responsive DEGs. Significantly enriched gene ontology (GO) terms were also sought based on “GO Term Finder” (http://www.yeastgenome.org/help/analyze/go-term-finder). The *B. oleracea* orthologues of *A. thaliana* genes were sought using the BioMart tool of EnsemblPlants release 46 [87]. The *A. thaliana* orthologues for the P-responsive DEGs in *B. oleracea* accessions C6 and F103 were identified using Ensembl. Of all 447 P-responsive DEGs in *B. oleracea*, 354 orthologues were identified in *A. thaliana* and the expression data for these was loaded into MapMan Version 3.5.0 BETA (https://mapman.gabipd.org/home) to analyse pathways affected by a reduced P supply. Line graphs were drawn in R, ggplot2 package [88], using MapMan outputs.

### Collection and metabolome profiling of root exudates

Since optimal root exudate collection by washing in trap solution is considered to be 2–6 h [59], the hydroponically-grown plants were placed (individually) in 100 mL of MilliQ water for 3 h (half of this time in the dark), frozen (at − 20 °C) and freeze dried (Alpha 2–4, Christ, Germany) for 3 days (− 50 °C, 0.12 mbar). However, this procedure yielded too small an amount of root exudates for all the analyses planned. Therefore, we repeated the experiment and exudates were collected over a period of twelve hours (half of this time in the dark), frozen and freeze dried. Still, the amount collected was not enough for the analyses planned. The results reported here came from a third attempt, when eighteen individuals of each accession from each treatment were placed in 100 mL of MilliQ water (without aeration), where they were kept for 24 h (half of this time in the dark) in the same greenhouse where the plants were grown. At the end of the collection period, in the morning, plants were removed from the solution, which was filtered (0.44 μm, Sigma-Aldrich), frozen and freeze dried. From the latter protocol, 0.9–3.8 μg of root exudates were collected for each treatment.

One half of each of the root exudate dry matter collected was used for the preliminary investigation of major functional groups using Fourier Transform Infrared (FTIR) spectroscopy, which was carried out at Beamline ID21 at the European Synchrotron Radiation Facility (ESRF) in Grenoble, France. Analysis was conducted on the Thermo Nicolet Nexus spectrometer (Artisan Technology Group, Champaign, IL, USA) associated with a SMART-orbit Attenuated Total Reflection (ATR) accessory as described previously [89]. All acquisitions were performed in the mid-IR spectral range (1700–800 cm^− 1^) in air.

On the second half of each of the root exudates collected, polar compounds were extracted and derivatised as described previously [90] and metabolite profiles were acquired using a gas chromatography-mass spectrometer (GC-MS; DSQII, Thermo-Finnigan, Hemel Hempstead, UK) system using a DB5-MSTM column (15 m × 0.25 mm × 0.25 μm; J&W, Folsom, CA, USA) as described by [91]. Compounds were identified by their mass spectrum and co-elution with authentic standards as determined using an in-house database. Data is presented as a relative value based upon the peak area of the specific ion used for quantification.

### Analysis of concentrations of elements in roots and shoots

Concentrations of P, sulphur (S), potassium (K), calcium (Ca), manganese (Mn), iron (Fe) and Zn were determined in oven-dried (70 °C for 3 days) roots (*n* = 5–7, composite samples from the initial 18 individuals used for the exudate collection) and shoots (*n* = 6) using inductively coupled plasma-mass spectrometry (ICP-MS) after microwave-assisted acid digestion as described by [92]. Phosphorus contents in root, shoots and whole plants were calculated by multiplying the P concentration in the respective organ with its corresponding biomass.

### High throughput root phenotyping (HTRP) experiment

Root system architecture was studied in a system similar to the one described by [85]. Seeds were placed on germination paper moistened with the same nutrient solutions used in the hydroponic experiments. Experiments were performed in 25 × 25 cm Petri dishes, which were arranged vertically in a growth chamber (maintained at, on average, 16 °C during 8 h night and 18 °C during the 16 h day). After 2 weeks of growth, roots were photographed and the length of the main root, and the number and length of lateral roots in each quartile of the main root length was determined using Fiji image processing software [93]. Three independent experiments were conducted, each having 8–11 replicates of each accession and treatment.

### Phosphorus distribution in roots

Plants from the HTRP experiments were used for the determination of P distribution in roots using micro-proton induced X-ray emission (micro-PIXE). Samples were prepared as described previously [94]. Pieces of roots below the first lateral root were placed into stainless-steel needles, cryo-fixed, cryo-sectioned to 50 μm thickness, freeze dried and sandwiched between two Pioloform foils (approx. 400 nm thickness; SPI supplies, West Chester, PA, USA) stretched over an aluminium frame. Micro-PIXE analysis was performed as described previously [95]. The micro-PIXE spectra were analysed and distribution maps were generated in the GeoPIXE II software package [96]. Because the distribution maps are quantitative each pixel provides information on the concentration of an element. Therefore, P concentrations were extracted from whole cross sections and from the endodermis of each section measured. In addition, the root cross-section diameter was determined. These calculations were performed using Fiji image processing software [93] as described previously [94].

### Statistical analysis

One-way analysis of variance (ANOVA) with accession as the independent variable and two-way ANOVA with accession and treatment as independent variables followed by a Holm-Sidak post-hoc test at *p* < 0.05 were employed when data were normally distributed. A non-parametric test (ANOVA on ranks, followed by Dunn’s test at *p* < 0.05) was employed in the case of non-parametric (lateral root length) data. The Student t-test (at *p* < 0.05) was used for comparisons of two groups. All statistical analyses were performed in SigmaPlot version 13.0 (Systat Software, San Jose, CA). The processing of ATR-FTIR spectra and the principal component analysis (PCA) for these spectra were performed using Orange 3.18 software [97, 98] and the workflow is shown in Additional file 1: Fig. S5. Data pre-processing for the PCA consisted of ATR correction using OMNIC Spectra (Thermo Fischer Scientific©) followed by rubber band baseline correction and vector normalisation.

## Supplementary information

**Additional file 1 Fig. S1.** Growth of *Brassica oleracea* accessions (C6 and F103) cultivated hydroponically for 2 weeks in solutions containing 0.025 mM P (*Low P*) and 0.25 mM P (*High P*). **Fig. S2.** Root (**a**), shoot (**b**) and plant (**c**) phosphorus (P) content of two *Brassica oleracea* accessions (C6 and F103) grown hydroponically for 2 weeks in solutions containing 0.025 mM P (*Low P*; LP) and 0.25 mM P (*High P;* HP). Data are means (*n* = 5–6) and standard errors. Asterisks indicate statistically significant differences (two-way analysis of variance followed by Holm-Sidak post hoc test at *P* < 0.05). **Fig. S3.** Root and shoot concentrations of calcium (Ca), sulphur (S), potassium (K), manganese (Mn) and iron (Fe) of two *Brassica oleracea* accessions (C6 and F103) grown hydroponically for 2 weeks in solutions containing 0.025 mM P (*Low P*; LP) and 0.25 mM P (*High P;* HP). Data are means (*n =* 5–6) and standard errors. Asterisks indicate statistically significant differences (two-way analysis of variance followed by Holm-Sidak post hoc test at *P <* 0.05). **Fig. S4.** Contributions of functional bands in the Fourier Transform Infrared spectra of root exudates collected from two *Brassica oleracea* accessions (C6 and F103) grown hydroponically for 2 weeks in solutions containing 0.025 mM P (*Low P*) and 0.25 mM P (*High P*) to the principal component one (PC1) and principal component two (PC2; PCA plot in Fig. 3b of the main text). **Fig. S5.** Workflow of the processing of the Fourier transform infrared spectroscopy spectra in Orange 3.18 software [1, 2].

**Additional file 2 Table S1.** Two-way analysis of variance (ANOVA) table with *p* values (those less than 5% are highlighted in bold) and means squares (in italics) for response variables in two *Brassica oleracea* accessions (C6 and F103) grown hydroponically for 2 weeks in solutions containing 0.025 mM P (*Low P*) or 0.25 mM P (*High P*); Zn, zinc. **Table S2.** Relative concentration of polar metabolites detected in root exudates of two *Brassica oleracea* accessions (C6 and F103) grown hydroponically for 2 weeks in solutions containing 0.025 mM P (*Low P*) or 0.25 mM P (*High P*). * indicates metabolites for which fold change could not be calculated, as it was not detected in one of the treatments in both accessions. **Table S3.** Two-way analysis of variance (ANOVA) table with *p* values (those less than 5% are highlighted in bold) and means squares (in italics) for main root length and ANOVA on Ranks for lateral root length along the main root length, divided into quartiles, in two *Brassica oleracea* accessions (C6 and F103) grown in the high throughput root phenotyping system with *Low phosphorus (P)* and *High P* treatments (four groups in total) for 2 weeks. **Table S4.** All differentially expressed P-responsive genes and their expression in FPKM, in roots of two *Brassica oleracea* accessions (C6 and F103) grown hydroponically for 2 weeks in solutions containing 0.025 mM P (Low P; LP) and 0.25 mM P (High P; HP). Differentially expressed genes were defined by comparing the gene expression in the Low P treatment with the gene expression in the High P treatment for each of the *B. oleracea* accession. **Table S5.** Gene ontology enrichment analysis for all differentially expressed genes in roots of two *Brassica oleracea* accessions (the C6 and the F103) grown in solutions containing 0.025 mM P (Low P) and 0.25 mM P (High P) for 2 weeks (*n* = 3 for each accession and each treatment). Bonferroni corrected *p*-values were used to determine significantly enriched categories and a corrected p-value of 0.05 was used as a cut-off. **Table S6.** P-responsive genes with orthologues in *Arabidopsis thaliana.* The table shows the gene expression of all P-responsive genes in the roots of two *Brassica oleracea* accessions (C6 and F103) grown hydroponically for 2 weeks in solutions containing 0.025 mM P (Low P) and 0.25 mM P (High P) which have *A. thaliana* ortholouges. **Table S7.** Shoot fresh weight, phosphorus (P) and zinc (Zn) concentrations and P efficiency ratio (calculated as Yield_low_ / (P_low_ × Yield_low_) or Yield_high_ / (P_high_ × Yield_high_)) of the two *Brassica oleracea* accessions (C6 and F103) grown in a peat-based compost amended with Low P (5.25 mg L^− 1^) and High P (15.75 mg L^− 1^) used in the experiments. Data for fresh weight, shoot P concentration and P efficiency ratio are from [1] and shoot Zn concentrations are from [2]; DM, dry matter.

## Data Availability

The datasets generated during and/or analysed during the current study are available from the corresponding author on reasonable request. RNAseq data can be found in Gene Expression Omnibus under the accession number GSE127467 (https://www.ncbi.nlm.nih.gov/geo/query/acc.cgi?acc=GSE127467).

## Abbreviations

AHA2: H^+^-exporting ATPase
ATR-FTIR: Attenuated Total Reflectance - Fourier Transform InfraRed
Ca: Calcium
DEG: Differentially expressed gene
Fe: Iron
FER1: Ferritin-1
FRD3: Ferric Reductase Defective
FRO2: Ferric Reduction Oxidase 2
GC-MS: Gas chromatography – mass spectrometry
GO: Gene ontology
HAK5: High Affinity potassium transporter HAK5
HTRP: High-throughput root phenotyping
ICP-MS: Inductively coupled plasma – mass spectrometry
IRT1: Iron-Regulated Transporter 1
K: Potassium
Micro-PIXE: Micro-particle induced – X-ray emission
Mn: Manganese
NAS: Nicotianamine Synthase
NRT2;1: Nitrate Transporter 2;1
P: Phosphorus
PHT: Phosphate Transporter
PC: Principal component
RSA: Root system architecture
S: Sulphur
Zn: zinc
