# Supplementary material for: Effect of phosphorus supply on root traits of two Brassica oleracea L. genotypes
Source: BMC Plant Biol. 2020 Aug 5;20:368. doi: 10.1186/s12870-020-02558-2 (PMC7404929; doi:10.1186/s12870-020-02558-2)
Supplement: Supplementary file 1 — Additional file 1 Fig. S1. Growth of Brassica oleracea accessions (C6 and F103) cultivated hydroponically for 2 weeks in solutions containing 0.025 mM P (Low P) and 0.25 mM P (High P). Fig. S2. Root (a), shoot (b) and plant (c) phosphorus (P) content of two Brassica oleracea accessions (C6 and F103) grown hydroponically for 2 weeks in solutions containing 0.025 mM P (Low P; LP) and 0.25 mM P (High P; HP). Data are means (n = 5–6) and standard errors. Asterisks indicate statistically significant differences (two-way analysis of variance followed by Holm-Sidak post hoc test at P < 0.05). Fig. S3. Root and shoot concentrations of calcium (Ca), sulphur (S), potassium (K), manganese (Mn) and iron (Fe) of two Brassica oleracea accessions (C6 and F103) grown hydroponically for 2 weeks in solutions containing 0.025 mM P (Low P; LP) and 0.25 mM P (High P; HP). Data are means (n = 5–6) and standard errors. Asterisks indicate statistically significant differences (two-way analysis of variance followed by Holm-Sidak post hoc test at P < 0.05). Fig. S4. Contributions of functional bands in the Fourier Transform Infrared spectra of root exudates collected from two Brassica oleracea accessions (C6 and F103) grown hydroponically for 2 weeks in solutions containing 0.025 mM P (Low P) and 0.25 mM P (High P) to the principal component one (PC1) and principal component two (PC2; PCA plot in Fig. 3b of the main text). Fig. S5. Workflow of the processing of the Fourier transform infrared spectroscopy spectra in Orange 3.18 software [1, 2]. [file 12870_2020_2558_MOESM1_ESM.docx]

**Additional file 1 for**

**Effect of phosphorus supply on root traits of two *Brassica oleracea* L. genotypes**

Paula Pongrac^1,2,*^, Hiram Castillo-Michel^3^, Juan Reyes Herrera^3^, Robert D. Hancock^4^, Sina Fischer^5^, Mitja Kelemen^2,6^, Jacqueline A. Thompson^1^, Gladys Wright^1^, Matevž Likar^7^, Martin R. Broadley^8^, Primož Vavpetič^2^, Primož Pelicon^2^ and Philip J. White^1,9,10^

^1^Ecological Science Group, The James Hutton Institute, Invergowrie, Dundee DD2 5DA, UK

^2^Jožef Stefan Institute, Jamova 39, SI-1000 Ljubljana, Slovenia

^3^European Synchrotron Radiation Facility, Grenoble, France

^4^Cell and Molecular Sciences, The James Hutton Institute, Invergowrie, Dundee DD2 5DA, UK

^5^Future Food Beacon of Excellence and the School of Biosciences, University of Nottingham, Nottingham, LE12 5RD, UK

^6^Jožef Stefan International Postgraduate School, Jamova 39, SI-1000 Ljubljana, Slovenia

^7^Biotechnical Faculty, University of Ljubljana, Jamnikarjeva 101, SI-1000 Ljubljana, Slovenia

^8^Plant and Crop Sciences Division, University of Nottingham, Loughborough, LE12 5RD, UK

^9^Distinguished Scientist Fellowship Program, King Saud University, Riyadh 11451, Saudi Arabia

^10^College of Resources and Environment, Huazhong Agricultural University, Wuhan 430070, China

*Corresponding author:

Jožef Stefan Institute, Jamova 39, SI-1000 Ljubljana, Slovenia

Tel: +386-51-222-963; Fax: +386 477 31 51

Email: [paula.pongrac@gmail.com](mailto:paula.pongrac@gmail.com)


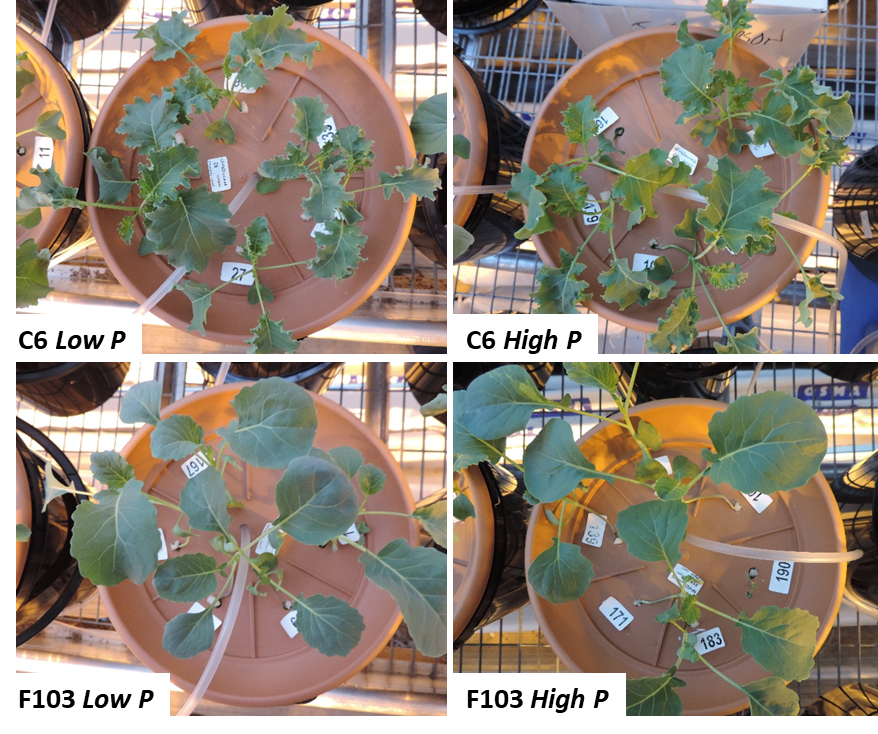


**Figure S1** Growth of *Brassica* *oleracea* accessions (C6 and F103) cultivated hydroponically for two weeks in solutions containing 0.025 mM P (*Low P*) and 0.25 mM P (*High P*).


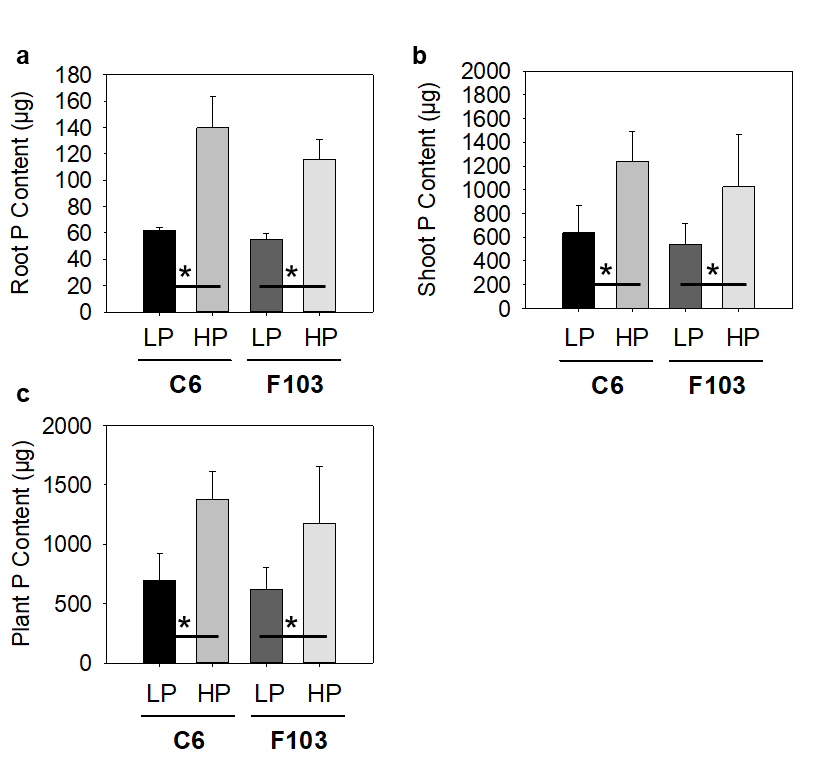


**Figure S2** Root (**a**), shoot (**b**) and plant (**c**) phosphorus (P) content of two *Brassica* *oleracea* accessions (C6 and F103) grown hydroponically for two weeks in solutions containing 0.025 mM P (*Low P*; LP) and 0.25 mM P (*High P;* HP). Data are means (n=5-6) and standard errors. Asterisks indicate statistically significant differences (two-way analysis of variance followed by Holm-Sidak post hoc test at P<0.05).


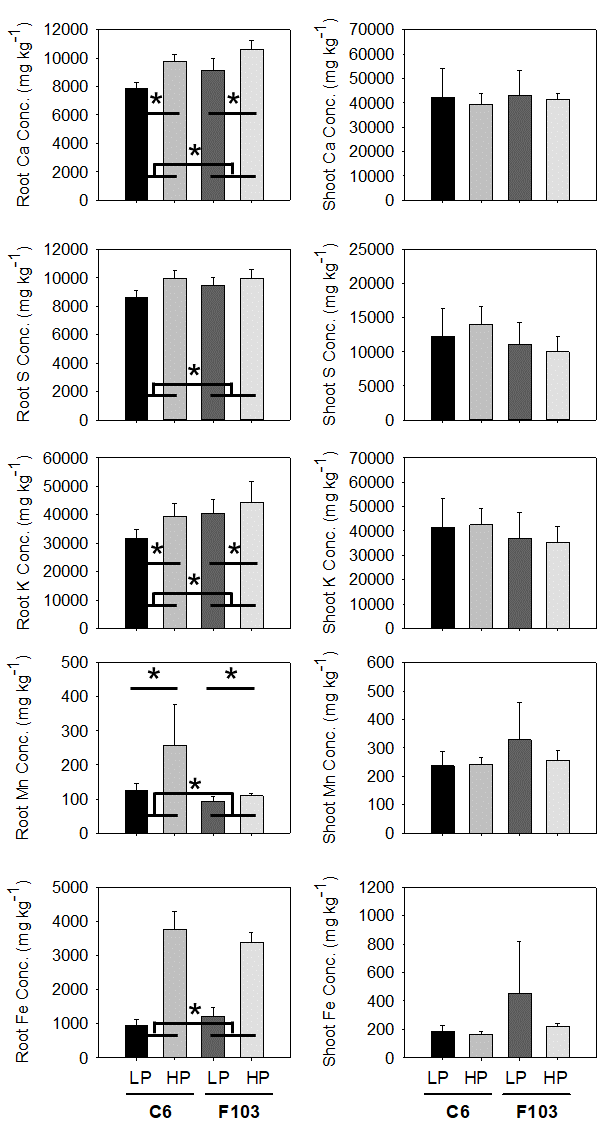


**Figure S3** Root and shoot concentrations of calcium (Ca), sulphur (S), potassium (K), manganese (Mn) and iron (Fe) of two *Brassica* *oleracea* accessions (C6 and F103) grown hydroponically for two weeks in solutions containing 0.025 mM P (*Low P*; LP) and 0.25 mM P (*High P;* HP). Data are means (n=5-6) and standard errors. Asterisks indicate statistically significant differences (two-way analysis of variance followed by Holm-Sidak post hoc test at P<0.05).


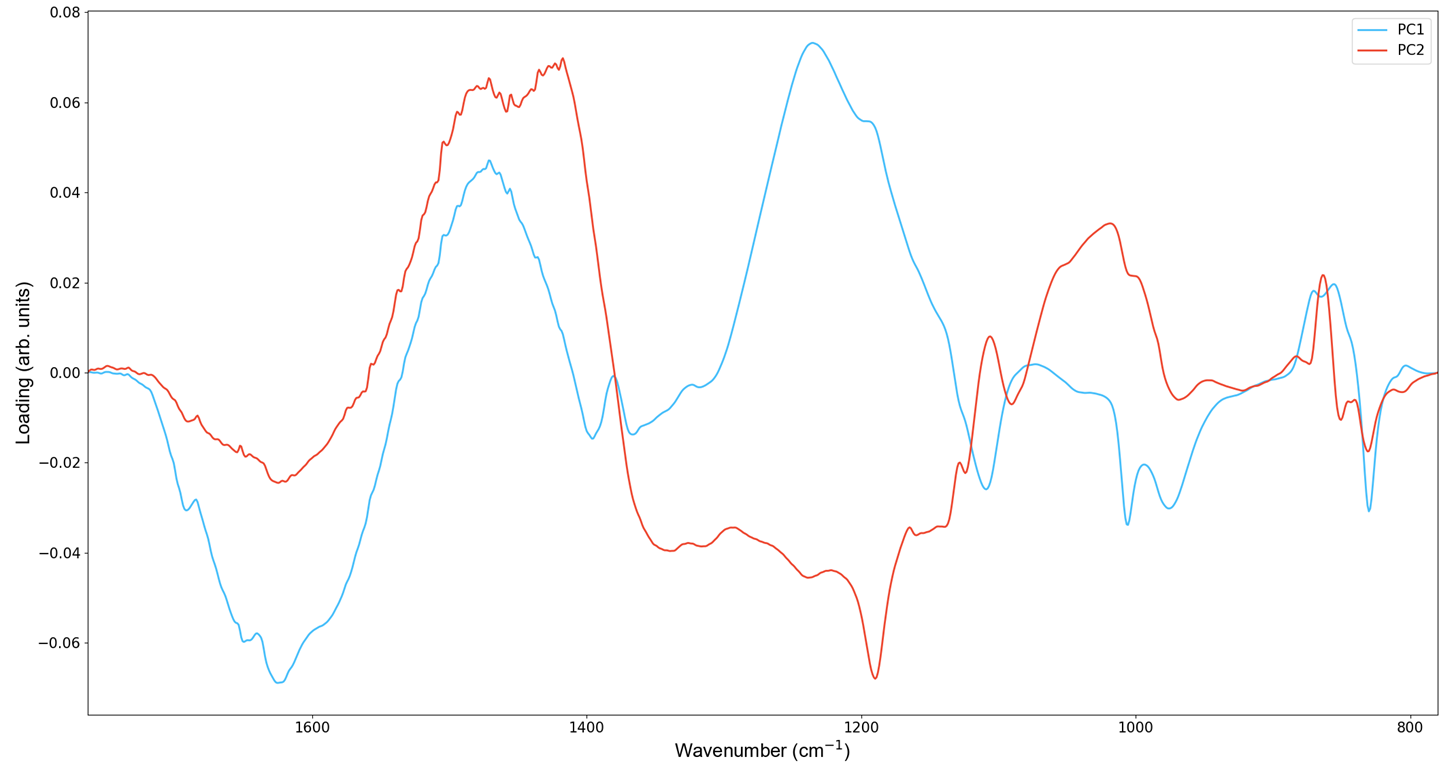


**Figure S4** Contributions of functional bands in the Fourier Transform Infrared spectra of root exudates collected from two *Brassica* *oleracea* accessions (C6 and F103) grown hydroponically for two weeks in solutions containing 0.025 mM P (*Low P*) and 0.25 mM P (*High P*) to the principal component one (PC1) and principal component two (PC2; PCA plot in **Fig. 3b** of the main text).


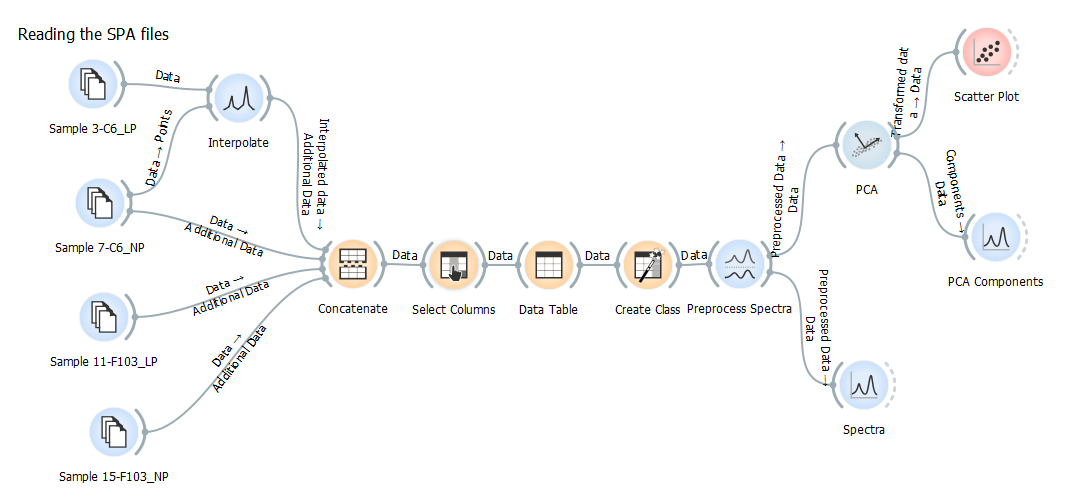


**Figure S5** Workflow of the processing of the Fourier transform infrared spectroscopy spectra in Orange 3.18 software [1, 2].

1. Demšar J, Curk T, Erjavec A, Hočevar T, Milutinovič M, Možina M, et al. Orange: Data mining toolbox in Python. J Mach Learn Res. 2013;14:23492353.

2. Toplak M, Birarda G, Read S, Sandt C, Rosendahl SM, Vaccari L, et al. Infrared Orange: Connecting hyperspectral data with machine learning. Synchrotron Radiat News. 2017;30:40–5. doi:10.1080/08940886.2017.1338424.
